# Vitamin D serum level predicts stroke clinical severity, functional independence, and disability—A retrospective cohort study

**DOI:** 10.3389/fnins.2022.951283

**Published:** 2022-07-27

**Authors:** Abdullah R. Alharbi, Amer S. Alali, Yahya Samman, Nouf A. Alghamdi, Omar Albaradie, Maan Almaghrabi, Seraj Makkawi, Saeed Alghamdi, Mohammad S. Alzahrani, Mohammed Alsalmi, Vardan T. Karamyan, Khalid Al Sulaiman, Ohoud Aljuhani, Faisal F. Alamri

**Affiliations:** ^1^King Abdullah International Medical Research Center, Jeddah, Saudi Arabia; ^2^College of Medicine, Umm Al-Qura University, Makkah, Saudi Arabia; ^3^Department of Pharmaceutics, College of Pharmacy, Prince Sattam Bin Abdulaziz University, Al-Kharj, Saudi Arabia; ^4^College of Medicine, King Saud Bin Abdulaziz University for Health Sciences, Jeddah, Saudi Arabia; ^5^Department of Basic Sciences, College of Science and Health Professions (KSAU-HS), King Saud Bin Abdulaziz University for Health Sciences, Jeddah, Saudi Arabia; ^6^Department of Medicine, College of Medicine, Al-Baha University, Al-Baha, Saudi Arabia; ^7^College of Medicine, King Abdulaziz University, Jeddah, Saudi Arabia; ^8^Department of Medicine, Ministry of the National Guard-Health Affairs, Jeddah, Saudi Arabia; ^9^Department of Clinical Pharmacy, College of Pharmacy, Taif University, Taif, Saudi Arabia; ^10^Department of Pharmaceutical Sciences, Jerry. H. Hodge School of Pharmacy, Texas Tech University Health Sciences Center, Amarillo, TX, United States; ^11^Center for Blood Brain Barrier Research, Jerry. H. Hodge School of Pharmacy, Texas Tech University Health Sciences Center, Amarillo, TX, United States; ^12^Department of Pharmaceutical Care, King Abdulaziz Medical City, Riyadh, Saudi Arabia; ^13^College of Pharmacy, King Saud Bin Abdulaziz University for Health Sciences, Riyadh, Saudi Arabia; ^14^King Abdullah International Medical Research Center, Riyadh, Saudi Arabia; ^15^Saudi Critical Care Pharmacy Research (SCAPE) Platform, Riyadh, Saudi Arabia; ^16^Department of Pharmacy Practice, King Abdulaziz University, Jeddah, Saudi Arabia; ^17^King Salman Center for Disability Research, Riyadh, Saudi Arabia

**Keywords:** stroke, vitamin D, 25(OH)D, modified Rankin scale (mRS), NIHSS score, disability, recovery, prevention

## Abstract

**Background:**

Stroke is a leading cause of mortality and disability and one of the most common neurological conditions globally. Many studies focused on vitamin D as a stroke risk factor, but only a few focused on its serum level as a predictor of stroke initial clinical severity and recovery with inconsistent results. The purpose of this study was to assess the relationship between serum vitamin D levels and stroke clinical severity at admission and functional independence and disability at discharge in Saudi Arabia.

**Methodology:**

A retrospective cohort study of adult ischemic stroke patients who had their vitamin D tested and admitted within 7 days of exhibiting stroke symptoms at King Abdulaziz Medical City (KAMC) Jeddah, Saudi Arabia. Based on vitamin D level, the patients were categorized into normal [25(OH)D serum level ≥ 75 nmol/L], insufficient [25(OH)D serum level is 50–75 nmol/L], and deficient [25(OH)D serum level ≤ 50 nmol/L]. The primary outcome was to assess the vitamin D serum level of ischemic stroke patients’ clinical severity at admission and functional independence at discharge. The National Institute of Health Stroke Scale (NIHSS) was used to assess the clinical severity, whereas the modified Rankin scale (mRS) was used to assess functional independence and disability.

**Results:**

The study included 294 stroke patients, out of 774, who were selected based on the inclusion and exclusion criteria. The mean age of the participants was 68.2 ± 13.4 years, and 49.3% were male. The patients’ distribution among the three groups based on their vitamin D levels is: normal (*n* = 35, 11.9%), insufficient (*n* = 66, 22.5%), and deficient (*n* = 196, 65.6%). After adjusting for potential covariates, regression analysis found a significant inverse relationship of NIHSS based on 25(OH)D serum level (beta coefficient: −0.04, SE: 0.01, *p* = 0.003). Patients with deficient serum vitamin D level also had significantly higher odds of worse functional independence in mRS score [OR: 2.41, 95%CI: (1.13–5.16), *p* = 0.023] when compared to participants with normal vitamin D level.

**Conclusion:**

Low vitamin D levels were associated with higher severity of stroke at admission and poor functional independence and disability at discharge in patients with acute ischemic stroke. Further randomized clinical and interventional studies are required to confirm our findings.

## Introduction

Stroke continues to be the world’s leading cause of long-term impairment of physical, cognitive, psychosocial, and other functions (Al Shoyaib et al., 2021a; Alamri et al., 2021). Nearly 85% of stroke survivors suffer paresis after stroke, and 50% of them continue living with motor impairments through the rest of their lives (Syeara et al., 2020). Several risk factors were found to be linked to stroke, including unmodifiable risk factors such as genetics, gender, and age, and modifiable risk factors such as hypertension, dyslipidemia, diabetes mellitus, atrial fibrillation, cigarette smoking, and sedentary lifestyle (Kuriakose and Xiao, 2020; Sayed et al., 2022). Given the limited therapeutic time window for improving stroke outcomes, identifying novel risk variables and severity predictors in ischemic stroke patients can aid in identification of patients that are at higher or lower risk of poor outcomes and allow stratification of patients for more personalized care and therapy (Kwakkel et al., 1996; Vijayan et al., 2019; Al Shoyaib et al., 2021b). Therefore, the discovery and validation of new predictive prognostic biomarkers is an active area of research, and some blood biomarkers and circulating molecules such as uric acid, interleukin 6, and vitamin D have already been introduced as predictors of stroke outcomes (Tu et al., 2013; Huang et al., 2016; Ng et al., 2017; Kamtchum-Tatuene and Jickling, 2019).

The physiological roles of vitamin D include calcium absorption from the gut, bone mineralization and growth, muscle function, and modulation of immunity (Lips, 2006; Bikle, 2009). In addition, recent studies indicate the role of vitamin D in cardiovascular disease prevention (Pilz et al., 2008). Low levels of 25-hydroxyvitamin D [25(OH)D, a vitamin D biomarker], have been linked to an elevated risk of coronary artery disease, ischemic heart disease, heart failure (HF), and stroke (Saponaro et al., 2019). Low vitamin D levels have also been linked to hypertension, dyslipidemia, and diabetes, all of which have an impact on cardiovascular and cerebrovascular events (Forman et al., 2007; Song et al., 2013; Lupton et al., 2016). Based on these studies, vitamin D deficiency is deemed to be a public health concern, especially for stroke patients.

In addition, several studies have investigated the role of vitamin D in stroke pathophysiology (Takeda et al., 2010; Wong et al., 2010; Balden et al., 2012). Vitamin D deficiency has been linked to increased post-stroke inflammatory activity by dysregulating the inflammatory response (Takeda et al., 2010; Wong et al., 2010; Balden et al., 2012). Vitamin D deficiency was associated with increased levels of interleukin-6 and high-sensitivity C-reactive protein in acute stroke patients (Wang et al., 2018). Also, vitamin D was shown to induce the expression of insulin-like growth factor 1 (IGF-1), a neuroprotective hormone that prevents axon and dendritic degeneration, and has antithrombotic properties (Turetsky et al., 2015; Yalbuzdag et al., 2015; Huang et al., 2016). Furthermore, vitamin D may also improve post-ischemic stroke vasodilation and neuronal survival by stimulating nitric oxide synthase (Turetsky et al., 2015; Yalbuzdag et al., 2015). These studies indicate a possible interaction between the level of vitamin D and stroke pathophysiology.

Previous experimental and observational studies have suggested that low vitamin D levels are independently associated with larger infarct volumes (Balden et al., 2012; Wang et al., 2014; Turetsky et al., 2015; Huang et al., 2016; Nie et al., 2017). Low levels of vitamin D have been identified in several epidemiological studies as a predictor of increased risk of stroke (Poole et al., 2006; Pilz et al., 2008; Chowdhury et al., 2012; Shi et al., 2020). Furthermore, decreased serum 25(OH)D levels in ischemic stroke patients were shown to independently predict stroke recurrence and mortality at 24 months (Qiu et al., 2017).

To date, limited studies have been done to investigate the impact of vitamin D level on stroke clinical severity and functional recovery with conflicting results (Bolland et al., 2010; Kuhn et al., 2013; Gupta et al., 2014; Wang et al., 2014; Park et al., 2015; Turetsky et al., 2015). Moreover, given the high prevalence (60%) of the Saudi Arabian population with vitamin D deficiency according to a recent meta-analysis (Al-Alyani et al., 2018). This study was conducted to assess the correlation between serum 25(OH)D levels and the clinical severity of ischemic stroke at admission and functional recovery at discharge in a cohort of patients in Saudi Arabia.

## Materials and methods

### Study design

A retrospective cohort study was conducted between June 2016 and July 2021 at King Abdulaziz Medical City (KAMC), Jeddah, Saudi Arabia. Admitted patients with acute or subacute ischemic stroke were included to assess the influence of vitamin D serum level on stroke clinical severity at admission and functional independence at discharge. Vitamin D was assessed by measuring the 25(OH)D total using chemiluminescence immunoassay (Abbott diagnostic analyzer: Architect i2000). The ischemic stroke clinical diagnosis was confirmed by brain Computed tomography (CT) and Magnetic resonance imaging (MRI) or both. The study was approved by King Abdullah International Medical Research Center (KAIMRC) Institutional Review Board in Riyadh-Saudi Arabia (Ref: RSS21J/003/06). Due to the retrospective observational nature of this study, the informed consent from the participants was waived.

### Study participants

**FIGURE 1:**
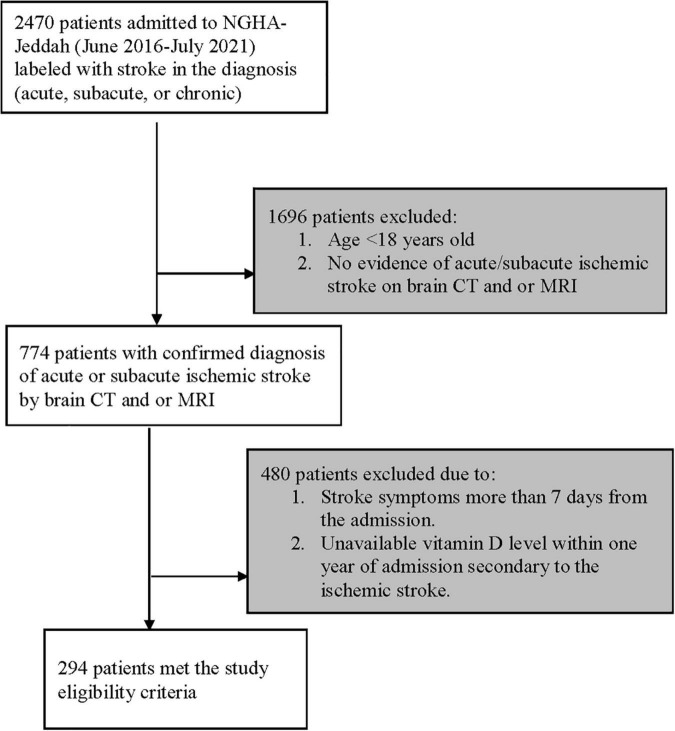
Flow diagram showing patients recruited with ischemic stroke. NGHA, National Guard Health Affairs; CT, Computerized tomography; MRI, Magnetic resonance imaging.

Patients were screened for eligibility using the electronic medical records system (BESTCare system 2.0) at the Ministry of National Guard-Health Affairs (MNGHA), Jeddah, Saudi Arabia. Patients with a confirmed clinical diagnosis of ischemic stroke within 7 days of symptoms onset were eligible for inclusion. Patients were excluded if aged less than 18 years old, or if vitamin D level measurements were unavailable within 1 year of admission secondary to ischemic stroke. All patients were followed until they were discharged from the hospital or died during the in-hospital stay, whichever occurred first (Figure 1).

### Outcomes

The primary outcome was to assess the correlation between patients’ vitamin D serum level and clinical severity of ischemic stroke at admission (NIHSS), whereas the secondary outcome was to evaluate the association of vitamin D level with functional independence and disability at discharge (mRS).

### Study setting

The study was conducted at KAMC—MNGHA. KAMC is a tertiary-care academic referral hospital with 509 bed capacity in Jeddah, Saudi Arabia. This center provides all types of care to all National Guard soldiers and their families, including primary health care and specialized tertiary care.

### Data collection

The collected data included demographic data (age, gender, BMI), comorbidities (hypertension, diabetes Mellitus, dyslipidemia, atrial fibrillation, dementia, and smoking), signs and symptoms of stroke (upper limb motor impairment, lower limb motor impairment, impaired consciousness, and aphasia), and complications [i.e., Pneumonia, deep vein thrombosis/pulmonary embolism (DVT/PE), hemorrhagic transformation, recurrent stroke, and death]. The presence of the aforementioned complications was accounted for if mentioned in the daily progress notes and validated by the attending physician. Diabetes mellitus, hypertension, and dyslipidemia were considered present if (1) a patient was on an antidiabetic or anti-hypertensive home medication, respectively, or (2) found in patient’s medical history, or finally, (3) found in laboratory assessment (comparing A1c and fasting glucose levels for diabetes; LDL, HDL, cholesterol, and triglyceride for dyslipidemia).

Vitamin D status was assessed by measuring 25-hydroxyvitamin D [25(OH)D] within 1 year of appearance of ischemic stroke symptoms. Eligible patients were categorized based on their 25-hydroxyvitamin D [25(OH)D] concentration into three groups: Normal (≥75 nmol/L), insufficient (50–75 nmol/L), and deficient (≤50 nmol/L) (Cuomo et al., 2019).

Stroke severity was assessed using the National Institutes of Health Stroke Scale (NIHSS) within 24 h of admission (Toth et al., 2017). Upon discharge, patients’ independence and degree of disability were assessed by mRS (Song et al., 2019). Available NIHSS and mRS scores were collected, whereas unavailable scores were calculated using cases’ descriptions in admission and discharge data. NIHSS and mRS scores were verified by a consultant neurologist.

### Statistical analysis

Univariable, bivariable, and multivariable analyses were performed. Univariable analysis was done to describe the demographic and clinical characteristics of study subjects. Frequencies and percentages were calculated for categorical variables, while means ± standard deviations (*SD*) or median with first and third quartiles (Q1, Q3) were calculated for numerical variables. Independent relationship with NIHSS (dependent variables) was assessed using simple and multiple linear regression. The functional outcome at discharge (mRS score) was dichotomized into good (mRS < 3) and poor (mRS ≥ 3). Simple and multiple binary logistic regression was used to identify factors associated with poor functional outcomes. Age, sex, and BMI were adjusted regardless of their bivariable association with each outcome. In addition, independent variables with *p*-values < 0.2 in the bivariable analysis (i.e., dyslipidemia, AF, and dementia) were included and adjusted for in the multivariable analysis. Linear regression coefficients (β) with standard error (SE) and odds ratios (OR) with 95% confidence intervals (CIs) were computed to determine the magnitude of associations of independent variables with the NIHSS and mRS. A *p*-value of less than 0.05 was considered statistically significant. All analyses were carried out using SAS University Edition (SAS Institute, Cary, NC, United States).

## Results

### Baseline characteristics of patients according to vitamin D status

A total of 2,470 patients were screened; 294 patients admitted with stroke were included in the present study based on the eligibility criteria. The mean age of included patients was 68.2 ± 13.4 years, and 49.3% were male. The female percentage in normal, insufficient, and deficient vitamin D level subgroups were 51.4, 53.1, and 49.7%, respectively. Hypertension was reported in 84%, diabetes mellitus 76.5%, and dyslipidemia 60.5% of the patients, and similar comorbidities percentage were observed within the vitamin D level sub-groups. The median time between stroke occurrence and vitamin D level measurement was 56 (Q1,Q3: 5,164) days. The median time between stroke occurrence and discharge was 30.5 days (Q1,Q3: 6,121) (Table 1).

**TABLE 1:** Characteristics of study subjects at admission according to vitamin D level.

	Total (*n* = 294)	Vitamin D level		
		
		Normal (*n* = 35)	Insufficient (*n* = 66)	Deficient (*n* = 193)
**Demographics**				
Sex, male, *n* (%)	145 (49.3)	17 (48.6)	31 (46.9)	97 (50.3)
Age, mean (*SD*)	68.2 ± 13.4	69.6 ± 12.7	68.4 ± 12.8	67.9 ± 13.8
BMI mean (*SD*)	29.4 ± 6.6	28.3 ± 4.2	28.6 ± 6.7	29.7 ± 6.9
**Presence of comorbidities**				
Hypertension (HTN), *n* (%)	247 (84)	28 (80)	57 (86.4)	162 (83.9)
Diabetes Mellitus (DM), *n* (%)	225 (76.5)	27 (77.1)	50 (75.8)	148 (76.7)
Dyslipidemia, *n* (%)	178 (60.5)	19 (54.3)	43 (66.2)	116 (60.1)
Atrial fibrillation, *n* (%)	47 (15.9)	5 (14.3)	12 (18.5)	30 (15.5)
Dementia, *n* (%)	18 (6.1)	2 (5.7)	3 (4.6)	13 (6.7)
Smoking, *n* (%)	39 (13.3)	5 (14.3)	7 (10.8)	27 (14.1)
**Severity of the stroke at admission**				
NIHSS, Median (Q1,Q3)	7 (4, 12)	5 (3, 9)	7 (4, 11)	8 (5, 13)
**Signs and symptoms**				
UL motor impairment, *n* (%)	206 (73.1)	23 (65.7)	40 (65.6)	143 (76.9)
LL motor impairment, *n* (%)	202 (71.9)	23 (65.7)	41 (67.2)	138 (74.6)
Impaired consciousness, *n* (%)	74 (26.2)	8 (24.2)	19 (29.2)	47 (25.4)
Aphasia, *n* (%)	56 (19.2)	6 (17.1)	12 (18.2)	38 (19.9)

Values are reported as number (%), mean ± standard deviation, or median (interquartile ranges). Missing: UL motor impairment (n = 12), LL motor impairment (n = 13), Impaired consciousness (n = 11).

BMI, body mass index; HTN, hypertension; DM, diabetes mellitus; NIHSS, The National Institutes of Health Stroke Scale; UL, upper limb; LL, lower limb.

The median NIHSS was 7 (Q1,Q3: 4,12). Stratified by vitamin D levels, patients with deficient vitamin D level had higher median NIHSS of 8 (Q1,Q3: 5,13) compared to those with insufficient [7 (Q1,Q3: 4,11)], or normal [5 (Q1,Q3: 3,9)] vitamin D levels (Table 1). Poor functional outcome (mRS ≥ 3) was reported in 178 subjects (60.5% of total subjects), out of which 16 subjects had normal vitamin D level (45.7% of normal vitamin D subjects), whereas 125 had deficient vitamin D level (64.8% of deficient vitamin D subject) (Table 2).

**TABLE 2:** Complications during hospital stay and functional independence at discharge according to vitamin D level.

	Total (*n* = 294)	Vitamin D level		
		
		Normal (*n* = 35)	Insufficient (*n* = 66)	Deficient (*n* = 193)
**Complications**				
Pneumonia, *n* (%)	37 (12.6)	4 (11.4)	9 (13.6)	24 (12.5)
DVT, *n* (%)	13 (4.5)	3 (8.6)	2 (3)	8 (4.2)
Hemorrhagic transformation, *n* (%)	18 (6.1)	1 (2.9)	3 (4.6)	14 (7.4)
Recurrent stroke, *n* (%)	50 (17.2)	10 (28.6)	13 (19.7)	27 (14.2)
Hospital mortality, *n* (%)	30 (10.2)	2 (5.7)	8 (12.1)	20 (10.4)
**Functional independence and disability at discharge**				
Poor (mRS ≥ 3), *n* (%)	178 (60.5)	16 (45.7)	37 (56.1)	125 (64.8)

Values are reported as number (%), mean ± standard deviation.

DVT, Deep vein thrombosis; mRS, the modified Rankin scale.

Upper and lower limb motor impairment were observed in 65.7% of the normal vitamin D group. Similar prevalence of upper (65.6%) and lower (67.2%) motor impairment in insufficient vitamin D group but these symptoms were higher in vitamin D deficient group in that 76.9% had upper limb and 74.6% had lower limb impairment (Table 1). The majority of death cases (28 out of 30) either belong to insufficient vitamin D group (*n* = 8, 12.1%) or deficient vitamin D group (*n* = 20, 10.4%) (Table 2).

### Association between the serum vitamin D level and national institute of health stroke scale

**FIGURE 2:**
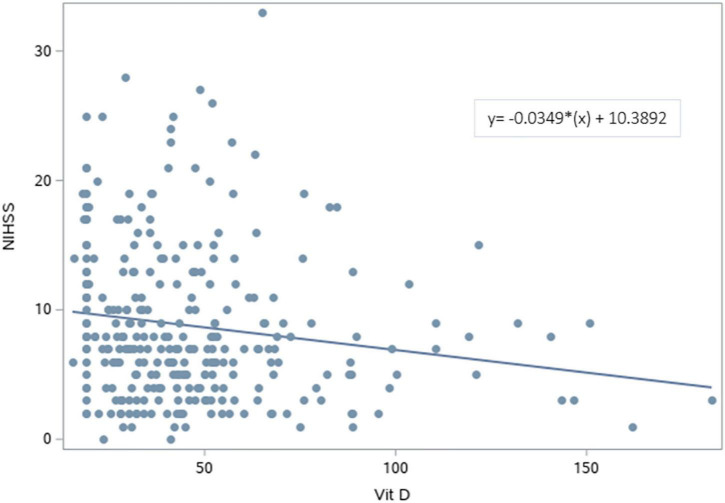
Scatterplot of the National Institutes of Health Stroke Scale vs. Vitamin D levels. A significant regression equation predicting NIHSS score based on vitamin D level was found (*F* = 7.44, *p* = 0.007). Patients predicted NIHSS score is equal to 10.3892–0.0349 (Vitamin D level) measured in nmol/L, with an *R*^2^ of 0.25.

The NIHSS was significantly higher for patients with deficient vitamin D level. The results revealed that lower vitamin D levels were significantly associated with higher NIHSS scores [β (SE) = −0.03 (0.01), *p* = 0.007] (Figure 2 and Table 3). After adjusting for age, sex, BMI, and variables with *p* < 0.2 in the bivariable analysis (dyslipidemia, AF, and dementia), vitamin D level remained a significant predictor of NIHSS score [β (SE) = −0.04 (0.01), *p* = 0.003].

**TABLE 3:** Simple and multiple linear regression analysis for association of independent variables with the NIH Stroke score.

Independent variables	Unadjusted		Adjusted	
		
	β (SE)	*P*	β (SE)	*P*
Sex, (female = 0, male = 1)	0.35 (0.69)	0.619	0.49 (0.69)	0.473
Age (years)	0.07 (0.03)	**0.007**	0.05 (0.03)	**0.043**
BMI (kg/m^2^)	−0.02 (0.05)	0.683	−0.01 (0.05)	0.795
HTN	0.39 (0.95)	0.682	−	−
DM	−0.27 (0.82)	0.739	−	−
Dyslipidemia	−1.08 (0.71)	0.126	−0.98 (0.69)	0.159
AF	1.86 (0.95)	**0.049**	1.52 (0.94)	0.107
Dementia	4.64 (1.42)	**0.001**	3.64 (1.45)	**0.013**
Smoking	0.44 (1.02)	0.669	−	−
Vitamin D (nmol/L)	−0.03 (0.01)	**0.007**	−0.04 (0.01)	**0.003**

β, regression coefficient; SE, standard error; BMI, body mass index; HTN, hypertension; DM, diabetes mellitus; AF, atrial fibrillation. p-values < 0.05 written in bold indicating statistical significant difference.

### Association between the serum vitamin D level and functional outcome at discharge

The simple and multiple logistic regression analyses results of functional outcomes are shown in Table 4. The simple logistic regression showed that patients with deficient vitamin D levels (OR = 2.18, 95% CI: 1.05–4.51) were significantly associated with poor functional outcomes. In the adjusted model, the odds of poor functional outcome (mRS ≥ 3) were more than two times greater among patients with deficient vitamin D levels than those with normal levels (adjusted OR = 2.41, 95% CI: 1.13–5.16).

**TABLE 4:** Simple and multiple logistic regression analysis for association of independent variables with poor functional outcome (mRS ≥ 3).

Independent variables	Unadjusted		Adjusted	
		
	OR (95% CI)	*P*	OR (95% CI)	*P*
**Sex**				
Female	Ref		Ref	
Male	1.01 (0.63–1.62)	0.959	1.05 (0.64–1.72)	0.854
Age (years)	1.01 (0.99–1.03)	0.08	1.01 (0.99–1.03)	0.195
BMI (kg/m^2^)	0.99 (0.96–1.03)	0.741	0.99 (0.95–1.03)	0.626
HTN	1.05 (0.56–1.98)	0.882	**−**	**−**
DM	0.91 (0.52–1.58)	0.73	**−**	**−**
Dyslipidemia	0.76 (0.47–1.23)	0.268	**−**	**−**
AF	2.1 (1.04–3.5)	**0.039**	1.99 (0.96–4.14)	0.064
Dementia	3.45 (0.98–12.21)	0.054	2.56 (0.69–9.49)	0.161
Smoking	0.83 (0.42–1.64)	0.589	**−**	**−**
**Vit D levels**				
Normal	Ref		Ref	
Insufficient	1.52 (0.67–3.45)	0.935	1.71 (0.73–4.01)	0.766
Deficient	2.18 (1.05–4.51)	**0.027**	2.41 (1.13–5.16)	**0.023**

OR, odds ratio; CI, confidence interval; BMI, body mass index; HTN, hypertension; DM, diabetes mellitus; AF, atrial fibrillation. p-values < 0.05 written in bold indicating statistical significant difference.

### Association between national institute of health stroke scale/modified rankin scale and comorbidities

The bivariable analysis showed that older age [β (SE) = 0.07 (0.03), *p* = 0.007] (Table 3), existence of atrial fibrillation [β (SE) = 1.86 (0.95), *p* = 0.049], or dementia [β (SE) = 4.64 (1.42), *p* = 0.001] were significant predictors of poorer NIHSS score. The presence of other comorbidities such as hypertension, diabetes mellitus, dyslipidemia, and smoking was not associated with NIHSS.

In addition, atrial fibrillation (OR = 2.1, 95% CI: 1.04–3.5) was associated with poor functional outcomes (Table 4). However, the presence of other comorbidities such as hypertension, diabetes mellitus, dyslipidemia, or smoking was not associated with poor functional outcomes at discharge.

## Discussion

The current study aimed to assess the relationship between serum 25(OH)D levels and stroke clinical severity at admission, and functional independence and disability at discharge. We found that low 25(OH)D levels were associated with worsening initial stroke severity and poor functional independence in ischemic stroke patients. These associations were further strengthened after the adjustment of common covariates such as age, BMI, dementia, and other comorbidities.

Only 11.9% of the patients in our study had sufficient vitamin D levels, while 66% of our participants had vitamin D deficiency. Other studies have found a high prevalence of vitamin D deficiency among stroke patients, which is consistent with our findings. According to a study conducted in Korea, 68% of stroke patients have vitamin D deficiency. Other studies conducted in China reported vitamin D deficiency in 68–78% of stroke patients (Tu et al., 2014; Wang et al., 2014; Park et al., 2015). This high prevalence of vitamin D deficiency can be related to a lack of sunlight exposure (Holick et al., 2012), limited mobility, and malnutrition, all of which are frequent in stroke patients and can lead to vitamin D deficiency (Bouziana and Tziomalos, 2011; Pilz et al., 2011; Holick et al., 2012). Also, this could explain the association of vitamin D deficiency with poor mRS as recovery after stroke is worse when there is limited mobility and malnutrition (Bouziana and Tziomalos, 2011; Pilz et al., 2011).

Few pre-clinical and clinical studies investigated the effects of Vitamin D deficiency on experimental stroke outcomes (Balden et al., 2012; Daubail et al., 2013; Tu et al., 2014; Sayeed et al., 2019; Rad et al., 2021). In animal models, experimental stroke studies found animals having vitamin D deficiency to be associated with more severe sensorimotor impairment, larger brain infarct size, and blood–brain barrier dysfunction (Balden et al., 2012; Sayeed et al., 2019). Moreover, the relationship between 25(OH)D levels and stroke clinical severity in patients with acute ischemic stroke has only been evaluated in few studies. Our findings extend on prior research indicating 25(OH)D is a predictive factor for initial stroke severity in acute ischemic stroke patients (Daubail et al., 2013; Tu et al., 2014; Rad et al., 2021). In contrast to these findings, other studies (Park et al., 2015; Hu et al., 2019) found no association between vitamin D levels and early stroke severity as measured by NIHSS score. Besides the differences in the study population and sample size, this inconsistency could be explained in part by the fact that some studies did not adjust for the typical covariant that might influence stroke severity.

Consistent with previous findings, our results show that vitamin D level is an independent predictor of poor functional outcome, measured by mRS (Tu et al., 2014; Wajda et al., 2019). On the contrary, Hu et al. (2019) demonstrated no significant correlation between vitamin D level and stroke functional outcome. Variation in study design, population, obtained, and adjusted crucial stroke covariates may account for those differences.

This observational study has several limitations for consideration. First, the retrospective design of this study limits our ability to address causality between vitamin D level and stroke severity. Second, this is a single-center study with a small sample size which could have underpowered the study. Third, despite our adjustment of many covariates in the multivariate analysis, there is potential for residual confounders (e.g., sun exposure, diet, physical activity, and parathyroid hormone level) that could affect the observed association. Lastly, administration of vitamin D supplement was not collected, measurement timing of vitamin D levels was not fixed, and some of the outcomes could not be assessed due to the retrospective nature (e.g., size of the infarction).

The current study comes in line with reports emphasizing low 25(OH)D can serve to predict the severity and recovery from ischemic stroke. Future studies need to focus on investigating whether vitamin D supplementation can reduce clinical severity or improve functional outcomes in acute ischemic stroke patients.

## Conclusion

Our findings indicate lower vitamin D level is associated with aggravating stroke clinical severity at admission and functional disability at discharge. Hence, vitamin D serum level may predict both stroke clinical severity at admission and functional disability at discharge. Future studies are required to understand the molecular pathology and whether vitamin D supplementation can be used in stroke prevention or treatment.

## Data availability statement

The datasets used and/or analyzed during this study are available from the corresponding author upon reasonable request.

## Ethics statement

The studies involving human participants were reviewed and approved by the King Abdullah International Medical Research Center Institutional Review Board, Riyadh, Saudi Arabia. Written informed consent for participation was not required for this study in accordance with the national legislation and the institutional requirements.

## Author contributions

All authors listed have made a substantial, direct, and intellectual contribution to the work, and approved it for publication.
